# Pharmacological mTOR targeting enhances the antineoplastic effects of selective PI3Kα inhibition in medulloblastoma

**DOI:** 10.1038/s41598-019-49299-3

**Published:** 2019-09-06

**Authors:** Frank Eckerdt, Jessica Clymer, Jonathan B. Bell, Elspeth M. Beauchamp, Gavin T. Blyth, Stewart Goldman, Leonidas C. Platanias

**Affiliations:** 1 Robert H. Lurie Comprehensive Cancer Center of Northwestern University, Chicago, IL USA; 2 Department of Neurological Surgery, Feinberg School of Medicine, Northwestern University, Chicago, IL USA; 3 Division of Hematology/Oncology/Neuro Oncology/Stem Cell Transplantation, Department of Pediatrics, Ann & Robert H. Lurie Children’s Hospital of Chicago, Chicago, IL USA; 4 Division of Hematology/Oncology, Department of Medicine, Feinberg School of Medicine, Northwestern University, Chicago, IL USA; 5 Medicine Service, Jesse Brown VA Medical Center, Chicago, IL USA; 6 Present Address: Dana-Farber/Boston Children’s Cancer and Blood Disorders Center, Boston, MA USA

**Keywords:** Paediatric cancer, Preclinical research, CNS cancer, Cancer stem cells

## Abstract

Despite recent advances in the treatment of medulloblastoma, patients in high-risk categories still face very poor outcomes. Evidence indicates that a subpopulation of cancer stem cells contributes to therapy resistance and tumour relapse in these patients. To prevent resistance and relapse, the development of treatment strategies tailored to target subgroup specific signalling circuits in high-risk medulloblastomas might be similarly important as targeting the cancer stem cell population. We have previously demonstrated potent antineoplastic effects for the PI3Kα selective inhibitor alpelisib in medulloblastoma. Here, we performed studies aimed to enhance the anti-medulloblastoma effects of alpelisib by simultaneous catalytic targeting of the mTOR kinase. Pharmacological mTOR inhibition potently enhanced the suppressive effects of alpelisib on cancer cell proliferation, colony formation and apoptosis and additionally blocked sphere-forming ability of medulloblastoma stem-like cancer cells *in vitro*. We identified the HH effector GLI1 as a target for dual PI3Kα and mTOR inhibition in SHH-type medulloblastoma and confirmed these results in HH-driven Ewing sarcoma cells. Importantly, pharmacologic mTOR inhibition greatly enhanced the inhibitory effects of alpelisib on medulloblastoma tumour growth *in vivo*. In summary, these findings highlight a key role for PI3K/mTOR signalling in GLI1 regulation in HH-driven cancers and suggest that combined PI3Kα/mTOR inhibition may be particularly interesting for the development of effective treatment strategies in high-risk medulloblastomas.

## Introduction

Medulloblastoma is one of the most common malignant brain tumours in paediatric patients accounting for approximately 20% of all paediatric brain tumours^1^. Though overall survival has improved with multimodal therapy including maximal surgical resection, radiation therapy, and multi-agent chemotherapy, this disease remains a significant cause of brain cancer morbidity and mortality^2^. Genomic studies have revealed four molecular subgroups; Wingless/Integrated (WNT), Sonic Hedgehog (SHH), Group 3, and Group 4^3^. These subgroups are biologically distinct and show differences in demographics, patterns of dissemination, and outcomes^4^. Of the 4 subtypes, SHH medulloblastoma is the most frequent in infants and adults^5,6^. Due to the very poor clinical outcome of some SHH medulloblastoma patients, the WHO has recently revised this classification, with new subclasses distinguishing between SHH, *TP53* wild-type and SHH, *TP53* mutant medulloblastoma^7^. Patients with SHH-driven medulloblastoma frequently exhibit either germline or somatic mutations and copy-number alterations in genes that regulate the Hedgehog (HH) signalling pathway such as *PTCH1*, *SUFU*, *SMO*, *GLI1*, *GLI2* and *MYCN*^8^. As a consequence, these genetic changes result in constitutive transcriptional activation of HH target genes, such as glioma-associated oncogene (GLI) transcription factors, that can promote cell proliferation and tumorigenesis. Aberrant HH signalling in cancer has led to the development of smoothened (SMO) inhibitors that target HH upstream components, thereby blocking target gene transcription^9^. However, SMO inhibitors in younger patients may result in complications due to the essential role of HH signalling during development^10,11^. Additionally, in humans and mice, SHH-type medulloblastomas acquire resistance to SMO inhibitors^9,12,13^. The aforementioned challenges outline major impediments for the utility of SMO inhibitors in paediatric cancers and have encouraged the search for alternative routes to target HH downstream effectors in SHH-driven paediatric cancers.

There is extensive crosstalk between the HH pathway and other pathways that can trigger GLI transcription factor activation in the absence of canonical HH signalling^14^. This has driven development of research seeking to identify putative oncogenic targets that are not part of the canonical HH pathway but stimulate GLI transcription factor activity through non-canonical routes. One such pathway is triggered by mechanistic target of rapamycin (mTOR)/S6K1 mediated phosphorylation of GLI1, resulting in GLI1 nuclear translocation and activation of HH target genes^15^. Additionally, crosstalk between the mTOR and HH pathways cooperates to promote mRNA translation through inhibition of eukaryotic translation initiation factor 4E-binding protein 1 (4EBP1) and stimulation of eukaryotic translation initiation factor 4E (eIF4E) expression^16,17^. Consistent with this, dual inhibition of the phosphatidylinositol 3-kinase (PI3K)/mTOR and HH pathways has been shown to suppress tumour growth and increase survival in a medulloblastoma flank xenograft mouse model^18^. Importantly, upregulation of components of the PI3K/AKT/mTOR pathway has been reported in SMO resistant medulloblastomas and concurrent combination of SMO antagonists with PI3K/mTOR inhibitors blocks tumour growth and prevents the development of resistance to SMO inhibitors^19,20^. This key role for mTOR has recently been corroborated by another study, reporting that loss of mTORC1 function blocks development of SHH-driven medulloblastoma^17^. Remarkably, pharmacologic mTOR inhibition was found to reduce tumour growth and increase survival in a SMO inhibitor resistant medulloblastoma transgenic mouse model^17^. Thus, accumulating evidence indicates that blocking components of the PI3K/mTOR pathway may be a promising approach for HH driven cancers.

Activation of PI3K signalling has been reported in medulloblastoma and may contribute to development and progression of this cancer^21–23^. Importantly, strategies targeting PI3K are not limited to SHH-driven medulloblastoma because pharmacological PI3K inhibition has also shown promise in *MYC*-driven Group 3 mouse models of medulloblastoma^23^. Elevated PI3K/AKT activity in glioma stem cells suggests generally important roles for this pathway in brain cancer stem cells (CSCs)^24^. Additionally, the PI3K/AKT pathway is activated after irradiation in nestin-positive cells in medulloblastoma mouse models, likely to promote survival and resistance of CSCs^21^. Accordingly, inhibition of PI3K signalling was shown to inhibit proliferation and promote differentiation of stem-like cancer cells in medulloblastoma^25^. Further, our own studies have demonstrated a key role for the PI3Kα catalytic isoform (p110α) in sphere forming medulloblastoma cells and showed disruption of cancer stem cell frequencies after *PIK3CA* knockdown^26^. Taken together, the PI3K pathway is attracting increasing recognition as a potential target to eradicate brain CSCs regardless of medulloblastoma subtype.

While the important contributions of PI3K/AKT activation for medulloblastoma development suggest that PI3K inhibitors might show promise for the treatment of this tumour, the narrow therapeutic window of pan-PI3K inhibitors has been discouraging^27^. Efforts to determine the discrete roles of PI3K isoforms and the clinical utility of isoform-selective inhibitors for PI3Ks indicate improved target selectivity, with lower toxicity^28^. Recent advances in the development of inhibitors of the alpha catalytic isoform suggest PI3Kα may be of particular interest for therapeutic approaches^29^. However, several studies have found that selective PI3Kα inhibition results in activation of the mTOR pathway to promote survival and resistance^30–33^.

Here, we sought to investigate the therapeutic effects of combined PI3Kα and mTOR inhibition in medulloblastoma and in particular the effects on the CSC population. We report evidence for a discrete role of the PI3Kα/mTOR pathway in SHH subtype medulloblastoma. We found dual PI3Kα and mTOR inhibition strongly reduced the amount of nuclear GLI1 protein in HH-driven medulloblastoma and similar results were observed in Ewing sarcoma, another HH-driven paediatric cancer. Finally, combined PI3Kα and mTOR targeting disrupted cancer stem cell frequencies *in vitro* and significantly inhibited tumour growth in a flank tumour xenograft mouse model  *in vivo*. These results suggest that selective PI3Kα targeting combined with mTOR inhibition has a therapeutic effect in medulloblastoma and potentially other HH-driven paediatric cancers.

## Results

### Alpelisib and OSI-027 inhibit PI3K/mTOR signalling and exhibit antineoplastic effects in medulloblastoma cells

**Figure 1:**
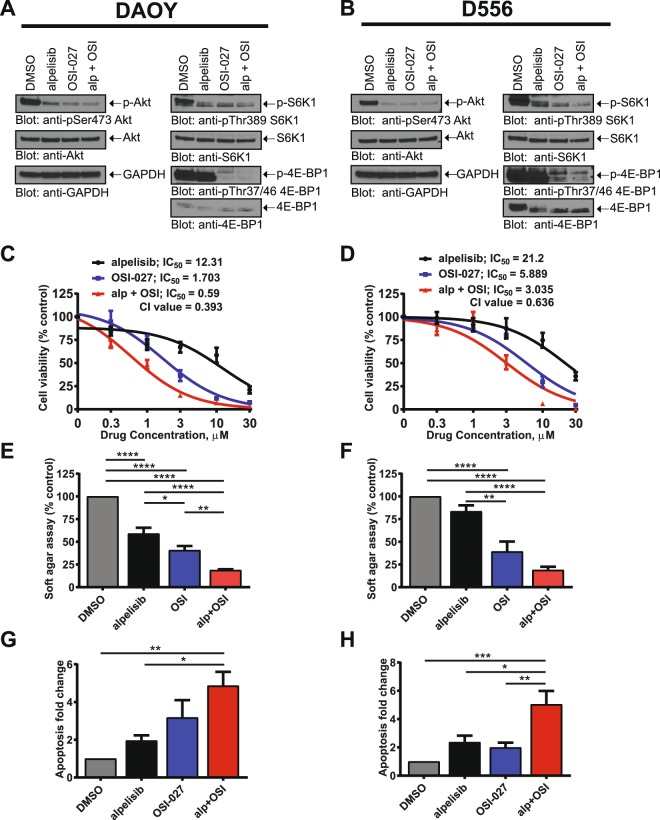
Alpelisib and OSI-027 inhibit PI3K/mTOR signalling and exhibit antineoplastic effects in medulloblastoma cells. (**A**,**B**) DAOY (**A**) or D556 (**B**) cells were treated with alpelisib (10 μM) and/or OSI-027 (10 μM) for 90 minutes and subjected to immunoblotting with antibodies against phospho-4EBP1^T37/46^ and phospho-S6K1^T389^. Membranes were stripped and reprobed with antibodies for 4EBP1, S6K1 and GAPDH. Lysates from the same experiment were run in parallel and subjected to immunoblotting with antibodies against phospho-AKT^S473^ followed by stripping and reprobing with antibodies for AKT. Blots were analysed by autoradiography. Uncropped blots are presented in the supplement. (**C,D**) DAOY (**C**) or D556 (**D**) cells in 96-well plates (2000 cells per well) were treated with alpelisib and/or OSI-027 at increasing concentrations for 5 days and cell viability was determined using the cell proliferation reagent, WST-1. Prism GraphPad 7.0 was used to determine IC_50_ values and CI values were calculated using Compusyn. Data represent means ± SEM of 3 independent experiments, each done in triplicate. (**E,F**) DAOY (**E**) or D556 (**F**) cells were seeded in soft agar in 96-well plates (1500 cells per well), treated with alpelisib (1 μM) and/or OSI-027 (1 μM). After 7 days, colony formation was quantified using the fluorescent CyQUANT GR Dye. Data represent means ± SEM of 5 independent experiments, each done in triplicate. Unpaired one-way ANOVA, *P ≤ 0.05, **P ≤ 0.01, ****P ≤ 0.0001. (**G,H**) DAOY (**G**) or D556 (**H**) cells were treated with alpelisib (10 μM) and/or OSI-027 (2 μM). After 2 days, apoptosis was assessed by costaining cells with propidium iodide (PI) and annexin V-FITC followed by flow cytometry analysis. Annexin V positive cells were quantified to determine total apoptosis. Data represent means ± SEM of 4 (DAOY) and 5 (D556) independent experiments. Unpaired one-way ANOVA, *P ≤ 0.05, **P ≤ 0.01, ***P ≤ 0.001.

Alpelisib is a PI3Kα-specific inhibitor with a tolerable safety profile^34^. However, several independent studies reported activation of resistance pathways, including mTORC1 signalling, that confer resistance to alpelisib in several solid tumours^30–33^. Thus, we sought to investigate the effects of simultaneous inhibition of PI3Kα and mTOR in DAOY and D556 medulloblastoma cells. We chose two cell lines that represent the highest risk category with very poor prognosis; DAOY representing the SHH, *TP53* mutated and D556 representing the Group 3 *MYC* amplified category^8,35,36^. Combined treatment with alpelisib and the catalytic mTOR inhibitor OSI-027 decreased phosphorylation of AKT (Ser^473^), S6K1 (Thr^389^), and 4E-BP1 (Thr^37/46^) (Fig. 1A,B). These results suggest that combined PI3Kα and mTOR inhibition potently blocks signalling of the PI3K-AKT-mTOR axis in medulloblastoma. This prompted us to study the biological effects of combined PI3Kα and mTOR inhibition. Initial experiments examined the dose response curves for inhibition of cell viability by alpelisib and OSI-027. In DAOY and D556 cells, combination of alpelisib with OSI-027 resulted in stronger suppression of cell viability as compared to either agent alone (Fig. 1C,D). In DAOY cells, the half maximal inhibitory concentration (IC_50_) decreased from 12.31 μM (alpelisib) and 1.703 μM (OSI-027) to 0.59 μM for the combination treatment (alpelisib and OSI-027) (Fig. 1C). In D556 cells, the IC_50_ decreased from 21.2 μM (alpelisib) and 5.889 μM (OSI-027) to 3.035 μM for the combination of alpelisib and OSI-027 (Fig. 1D). We next calculated the combination index (CI) for this drug combination. The CI defines additive effects (CI = 1), synergism (CI < 1) or antagonism (CI > 1)^37^. The combination of alpelisib with OSI-027 resulted in synergistic effects in both cell lines with CI values of 0.393 for DAOY and 0.636 in D556 cells, respectively. These findings are consistent with potent synergistic inhibitory effects in both cell lines, with such synergism being more potent in SHH-driven DAOY cells as compared to D556 cells that represent Group 3 medulloblastoma. In further studies, we found growth of colonies in soft agar was also potently inhibited by the combination of alpelisib and OSI-027 (Fig. 1E,F). Additionally, the combination of the two agents increased the rate of apoptosis substantially more than either agent alone (Fig. 1G,H). Together, these results suggest that catalytic mTOR inhibition strongly enhances the antineoplastic effects of selective PI3Kα inhibition in medulloblastoma cells.

### *PIK3CA* expression is elevated in the SHH-subgroup and correlates with *GLI1* expression in medulloblastoma

**Figure 2:**
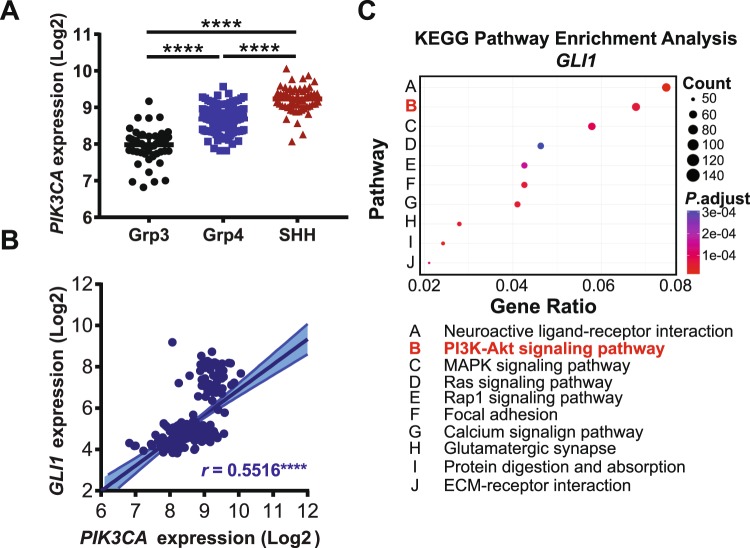
*PIK3CA* expression is elevated in the SHH-subgroup and correlates with *GLI1* expression in medulloblastoma. (**A**) *PIK3CA* expression was analyzed in different medulloblastoma subgroups from the Northcott_2012 dataset, downloaded from the GlioVis portal (http://gliovis.bioinfo.cnio.es/) and subjected to analysis in GraphPad Prism 7.0. Unpaired one-way ANOVA, ****P ≤ 0.0001. (**B**) Gene expression data from the Northcott_2012 dataset were downloaded from the GlioVis portal and GraphPad Prism 7.0 was used for correlation analysis to compare *PIK3CA* expression with *GLI1*. Pearson’s correlation coefficient is shown, ****P ≤ 0.0001. (**C**) Medulloblastoma patient data from the Northcott_2012 dataset were subjected to KEGG pathway enrichment analysis for *GLI1* using the GlioVis portal (http://gliovis.bioinfo.cnio.es/). Bubble chart shows enrichment for the given pathway, where each point represents the enrichment level, the colour corresponds to the adjusted *P*-value (*P*.adjust), and the size corresponds to the number of genes enriched (Count). Y-axis label represents pathway (see bottom panel for detailed name of pathways), and X-axis label represents enrichment factor (amount of differentially expressed genes enriched in the pathway over amount of all genes in background gene set).

Recently, at least four major subtypes of medulloblastoma have been described^3,38^. We next sought to investigate whether distinct subgroups may show different dependencies on the PI3Kα signalling axis. Analysis using the Northcott dataset^39^ revealed that *PIK3CA* was most highly expressed in SHH subtype medulloblastoma (Fig. 2A). *PIK3CA* expression was also found to positively correlate with expression of the HH effector transcription factor glioma-associated oncogene 1 (GLI1) (Fig. 2B). Furthermore, pathway enrichment analysis indicates that *GLI1* expression is significantly associated with the “PI3K-AKT signalling pathway” in medulloblastoma (Fig. 2C). Together, these data indicate a correlation between *PIK3CA* expression and *GLI1* in medulloblastoma and raise the possibility of a functional relationship between PI3Kα signalling and the GLI1 transcription factor in HH driven cancers.

### Dual PI3Kα and mTOR inhibition decrease the amount of nuclear GLI1 and exhibit potent antineoplastic effects in Ewing sarcoma cells

**Figure 3:**
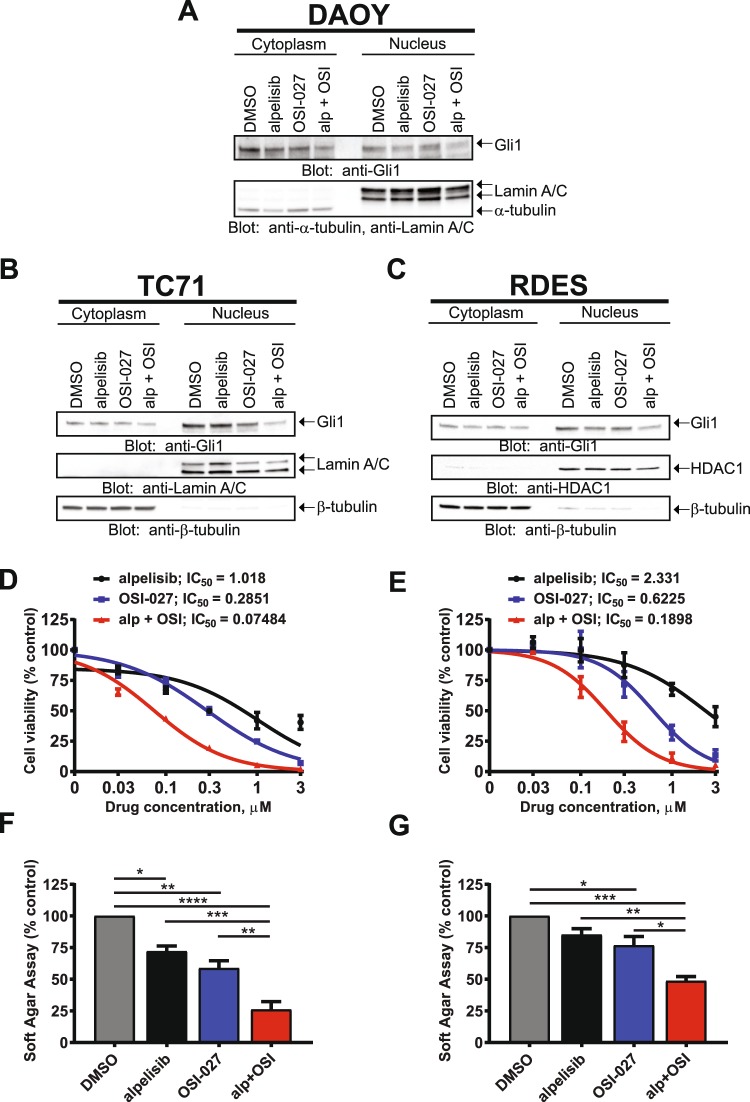
Dual PI3Kα and mTOR inhibition decreases the amount of nuclear GLI1 and exhibit potent antineoplastic effects in Ewing sarcoma cells. (**A**) DAOY medulloblastoma cells were treated with alpelisib (10 μM) and/or OSI-027 (5 μM) for 12 hours. After cell fractionation, nuclear and cytoplasmic extracts were subjected to immunoblotting using antibodies against GLI1, Lamin A/C and tubulin. Membranes were analysed using the ChemiDoc MP Imaging System. (**B**) TC71 Ewing Sarcoma cells were treated with alpelisib (0.15 μM) and/or OSI-027 (0.15 μM) for 24 hours and subjected to cell fractionation and immunoblotting using antibodies against GLI1, Lamin A/C and tubulin. Membranes were analysed using the ChemiDoc MP Imaging System. (**C**) RDES Ewing Sarcoma cells were treated with alpelisib (0.15 μM) and/or OSI-027 (0.15 μM) for 24 hours and subjected to cell fractionation and immunoblotting using antibodies against GLI1 and Lamin A/C. Membranes were stripped and reprobed with antibodies against HDAC1. Membranes were analysed using the ChemiDoc MP Imaging System. Uncropped blots for (**A–C**) are presented in the supplement. (**D,E**) TC71 (**D**) or RDES (**E**) Ewing Sarcoma cells were treated with alpelisib and/or OSI-027 at increasing concentrations for 5 days and cell viability was determined using the cell proliferation reagent, WST-1. Prism GraphPad 7.0 was used to determine IC_50_ values. Data represent means ± SEM of 3 independent experiments, each done in triplicate. (**F,G**) TC71 (F) or RDES (**G**) Ewing Sarcoma cells were seeded in soft agar in 96-well plates (5000 cells per well), treated with alpelisib (0.3 μM) and/or OSI-027 (0.3 μM). After 10 days, colony formation was quantified using the fluorescent CyQUANT GR Dye. Data represent means ± SEM of 3 independent experiments, each done in triplicate. Unpaired one-way ANOVA, *P ≤ 0.05, **P ≤ 0.01, ***P ≤ 0.001, ****P ≤ 0.0001.

GLI1 phosphorylation by the mTOR/S6K1 pathway promotes GLI1 nuclear localization and transcriptional activity, providing a mechanism for smoothened (SMO)-independent GLI1 activation^15^. We examined the effects of PI3Kα and mTOR inhibition on GLI1 nuclear accumulation in SHH-driven medulloblastoma using nuclear/cytoplasmic fractionation of DAOY cells with Lamin A/C and tubulin as nuclear and cytoplasmic loading controls, respectively. Combination of alpelisib and OSI-027 resulted in a decrease of nuclear GLI1 protein amounts, which was more pronounced as compared to single agent treatment (Fig. 3A). Next, we sought to investigate whether this GLI1 regulation by PI3Kα/mTOR is also evident in other HH-driven paediatric cancers. GLI1 transcription is directly induced by Ewing sarcoma breakpoint region 1-Friend leukemia virus integration 1 (EWS-FLI1), which promotes carcinogenesis of Ewing sarcoma Family of Tumors (ESFT)^40^. Therefore, we employed two Ewing sarcoma cell lines (TC71 and RDES) to examine GLI1 cytoplasmic/nuclear distribution in this cancer. Importantly, in TC71 and RDES Ewing sarcoma cells, combined PI3Kα and mTOR inhibition resulted in reduced nuclear GLI1 (Fig. 3B,C). The reduction of GLI1 protein in the nucleus after dual PI3Kα and mTOR inhibition indicates a requirement for PI3Kα and mTOR activities to sustain nuclear GLI1 in HH-driven cancers. HH-driven cancers strongly depend on nuclear GLIs for transcriptional activation of GLI target genes to promote proliferation and tumorigenesis^41^. Therefore, reduced nuclear GLI protein may contribute to suppression of tumour cell proliferation. In line with this, TC71 and RDES Ewing sarcoma cells exhibited high sensitivity to alpelisib and OSI-027 with IC_50_ values in the nanomolar range for the alpelisib/OSI-027 combination (TC71: 74 nM; RDES: 189 nM) (Fig. 3D,E). Additionally, dual PI3Kα and mTOR inhibition significantly reduced anchorage-independent growth of TC71 and RDES cells in soft agar as compared to either drug alone (Fig. 3F,G). These data support a role for PI3Kα and mTOR activities in maintaining nuclear GLI1 protein and inhibition of PI3Kα and mTOR disrupts the accumulation of GLI1 in the nucleus which may, at least in part, contribute to greatly reduced cell viability and colony formation in HH-driven paediatric cancers.

### Inhibition of PI3Kα or *PIK3CA* knockdown reduces sphere formation and disrupts medulloblastoma stem cell frequencies when combined with pharmacologic mTOR inhibition

**Figure 4:**
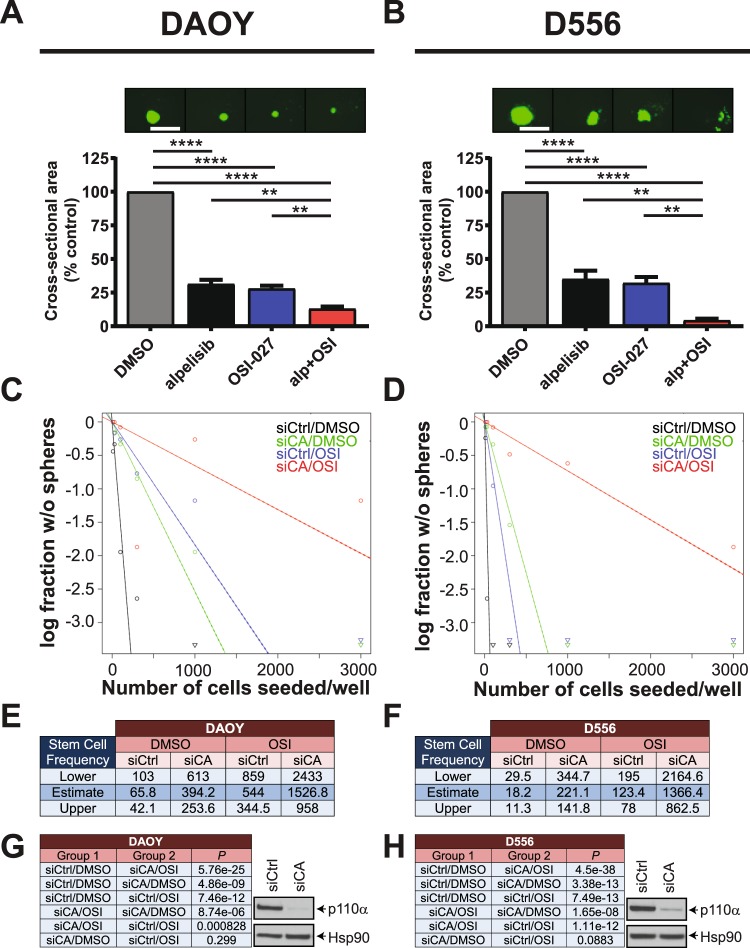
Inhibition of PI3Kα or *PIK3CA* knockdown reduces sphere formation and disrupts medulloblastoma stem cell frequencies when combined with pharmacologic mTOR inhibition. (**A,B**) DAOY (**A**) or D556 (**B**) cells were grown as spheres in CSC medium for 7 days. Spheres were dissociated and seeded at 500 cells/well into round-bottom 96-well plates in the presence of alpelisib (5 μM) and/or OSI-027 (1 μM). After 7 days, spheres were stained with acridine orange and imaged to determine cross-sectional area. Data represent means ± SEM of 3 independent experiments, each done in triplicate. Unpaired one-way ANOVA, **P ≤ 0.01, ****P ≤ 0.0001. Representative images are shown in the top panels. Scale bar, 1,000 μm. (**C**,**D**) *In vitro* ELDA after *PIK3CA* knockdown in combination with mTOR inhibition. DAOY (**C**) and D556 (**D**) cells were transfected with control siRNAs (siCtrl) or siRNAs targeting *PIK3CA* (siCA). After 2 days, cells were dissociated with trypsin and seeded in 3–5 technical replicates (n = 3) into round-bottom 96-well plates by forward- and side scatter, single-cell sorting at densities of 10, 30, 100, 300, 1,000 or 3,000 cells per well. Cells were treated with DMSO or OSI-027 (2 μM). After 7 days, neurospheres were stained with acridine orange and imaged using a Cytation 3 Cell Imaging multi-Mode Reader with a 4x objective. Neurospheres with a diameter of ≥100 μm were scored positive for ELDA analysis (http://bioinf.wehi.edu.au/software/elda/). (**E,F**) Stem cell frequencies of medulloblastoma stem-like cancer cells for DAOY (**E**) or D556 (**F**) were estimated as the ratio 1/*x* with the top and bottom 95% confidence intervals, where 1 = stem cell and *x* = all cells. (**G,H**) *P* values from χ^2^ analyses are shown for DAOY (**G**, left panel) and D556 (**H**, left panel). Whole cell lysates of DAOY (**G**, right panel) and D556 (**H**, right panel) were subjected to immunoblotting using antibodies against p110α to monitor knockdown of *PIK3CA*. Membranes were stripped and reprobed with antibodies against HSP90. Blots were analysed by autoradiography. Uncropped blots are presented in the supplement.

Next we sought to test the effects of combined PI3Kα and mTOR inhibition on stem-like cancer cells grown in 3-D. Ewing sarcoma spheroids may not represent a reliable system to study stem-like cancer cell biology^42^. By contrast, we and others have shown that medulloblastoma cells grown under cancer stem cell conditions form 3-D neurospheres that adopt cancer stem cell characteristics^26,43–45^. We have also demonstrated a key role for the alpha catalytic PI3K isoform in medulloblastoma sphere-forming cells^26^. Therefore, we used medulloblastoma neurospheres grown in 3-D to study sphere-forming ability of stem-like cancer cells. Neurosphere growth was potently inhibited by alpelisib and OSI-027 and combination of both inhibitors resulted in significantly stronger blockade of neurosphere growth than either drug alone (Fig. 4A,B). These results indicate greatly reduced sphere-forming ability of medulloblastoma neurospheres after dual PI3Kα and mTOR inhibition.

We have previously investigated distinct roles for Class I_A_ PI3K isoforms and reported that expression of *PIK3CA* correlates with the expression of pluripotency/stem cell markers in medulloblastoma suggesting an important role for PI3Kα in the biology of medulloblastoma CSCs^26^. In line with this, we demonstrated an essential role for *PIK3CA* but not *PIK3CB* or *PIK3CD* in maintaining self-renewal capacities of stem-like cancer cells as judged by disruption of stem cell frequencies after knockdown of Class I_A_ catalytic isoforms in extreme limiting dilution analysis (ELDA)^26^. However, evidence from breast cancer suggests a role for mTORC1 in resistance to selective PI3Kα inhibition and thus a requirement for mTORC1 blockade to enhance efficacy of PI3Kα inhibition^30,32^. Also, our previous work has demonstrated that the effects of *PIK3CA* knockdown or pharmacological PI3Kα inhibition can be enhanced by concomitant inhibition of pro-survival pathways in medulloblastoma^26^. Therefore, we used RNA interference (RNAi) to determine a possible role for mTOR activity in maintaining CSC frequencies after *PIK3CA* knockdown. *PIK3CA* knockdown strongly reduced the proportion of cells with self-renewal capacity in both DAOY (Fig. 4C) and D556 (Fig. 4D) cells. Importantly, the combination of OSI-027 with *PIK3CA* knockdown significantly reduced stem cell frequencies over either *PIK3CA* knockdown or OSI-027 single agent alone. Specifically, stem cell frequencies in DAOY dropped from 1 in 394.2 cells for *PIK3CA* knockdown or 1 in 544 for OSI-027 to 1 in 1526.8 for the combination *PIK3CA* knockdown and OSI-027 (Fig. 4E). Similar results were observed for D556 where stem cell frequencies decreased from 1 in 221.1 for *PIK3CA* knockdown or 1 in 123.4 for OSI-027 to 1 in 1366.4 for the combination (Fig. 4F). Chi-square analysis revealed these changes in stem cell frequencies were highly significant (Fig. 4G,H left panels). Knockdown of p110α protein was monitored by western blot analysis (Fig. 4G,H right panels). These results indicate that pharmacologic mTOR inhibition enhances the disruptive effects of *PIK3CA* knockdown on stem cell frequencies and suggest dual inhibition of PI3Kα and mTOR is required to efficiently block self-renewal capabilities of medulloblastoma CSCs.

### Dual PI3Kα and mTOR inhibition reduces tumour growth in a medulloblastoma flank tumour xenograft

**Figure 5:**
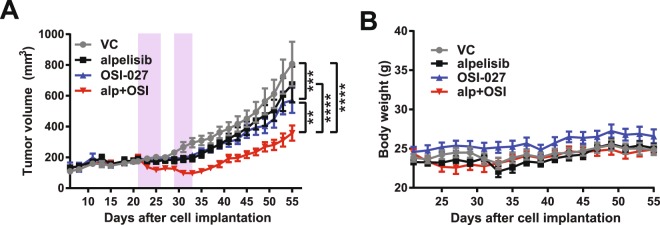
Dual PI3Kα and mTOR inhibition reduces tumour growth in a medulloblastoma flank tumour xenograft. (**A**) Tumour volumes from a flank xenograft mouse model are shown. Nude mice were injected subcutaneously into the left flank with DAOY cells (5 × 10^6^ cells/mouse). Once mice showed palpable tumours, mice were randomized into vehicle control (VC; n = 10), alpelisib (50 mg/kg; n = 9), OSI-027 (25 mg/kg; n = 9) or combination (alpelisib and OSI-027; n = 10) groups. Mice were treated by oral gavage for 5 days, then 2 days rest followed by another 4 days of treatment (indicated by purple boxes). Two-way ANOVA for day 55; **P ≤ 0.01, ***P ≤ 0.001, ****P ≤ 0.0001. (**B**) Body weight of mice from flank tumour xenograft experiment. Mouse body weight from experiment in A was recorded every other day throughout the study.

Given the potent antineoplastic effects of alpelisib and OSI-027 *in vitro*, we next sought to investigate the effects of dual PI3Kα and mTOR inhibition *in vivo*. As DAOY cells are representatives of high-risk SHH-driven medulloblastoma^35^, we used a DAOY medulloblastoma flank tumour xenograft mouse model to study the antitumor activity of combined PI3Kα and mTOR inhibition. DAOY cells in matrigel were injected subcutaneously in the flanks of nude mice and when tumours were palpable, mice were treated with vehicle controls (VC), alpelisib, OSI-027 or the alpelisib/OSI-027 combination (5 days treatment, 2 days rest followed by another 4 days treatment). While each drug moderately reduced the rate of tumour growth relative to vehicle control treated mice, the alpelisib/OSI-027 combination significantly reduced tumour growth as compared to either drug alone (Fig. 5A). The absence of obvious changes in body weight suggests that the combination of alpelisib with OSI-027 at these concentrations was well tolerated while still reducing tumour growth (Fig. 5B).

## Discussion

Current treatments have produced promising results for many patients diagnosed with medulloblastoma, but not for patients with high-risk tumours. Due to the poor prognosis of some SHH medulloblastomas, the 2016 WHO consensus conference has reclassified subgroups into: WNT, SHH and *TP53* wild-type, SHH and *TP53* mutant, and non-WNT/non-SHH (encompassing Group 3 and Group 4 within this subgroup)^7^. Together with Group 3, metastatic and Group 3 *MYC* amplified medulloblastoma, SHH, *TP53* mutant medulloblastoma has been identified as the highest risk category with very poor prognosis, as evident from 5-year overall survival rates of only 41%^8,36^. This very poor prognosis resulted in listing these patients with SHH, *TP53* mutations as their own category by the WHO^7,46^.

In the current study, we investigated the effects of combined PI3Kα and mTOR inhibition in two of the highest risk category medulloblastoma cells, with DAOY cells representing the SHH, *TP53* mutant and D556 cells representing the Group 3 *MYC* amplified categories^35^. The p110α isoform is highly expressed or activated among medulloblastoma patient samples^47^, and several investigations have been reporting promising results for targeting PI3K signalling in the SHH-driven subgroup^17–19^ and the *MYC*-driven Group 3^23,48^. We utilized the PI3Kα selective inhibitor alpelisib, because the alpha catalytic isoform p110α exerts important roles in medulloblastoma^47,49^ and is the only Class I_A_ PI3K essential for sphere-forming ability and stem cell frequencies in medulloblastoma stem-like cancer cells^26^. Isoform-selective PI3Kα inhibitors are now extensively exploited because of higher efficacy and lower toxicity as compared to pan-PI3K inhibitors^29^. Alpelisib is one such PI3Kα selective inhibitor with encouraging results in early phase clinical trials and has a suitable safety profile^34^. However, it has been reported that tumours develop resistance to alpelisib by activation of the mTOR pathway^30,31,33^. Therefore, we reasoned that alpelisib should be tested in combination with pharmacological mTOR inhibitors. We found that the combination of alpelisib with the catalytic mTOR inhibitor OSI-027 efficiently blocked signalling of the PI3K/AKT/mTOR pathway, exhibited potent antineoplastic effects, induced apoptosis and inhibited sphere formation. Additionally, knockdown of *PIK3CA* (encoding p110α) in combination with OSI-027 disrupted stem cell frequencies. Thus, dual PI3Kα and mTOR inhibition is a comprehensive strategy that includes targeting the CSC population and therefore shows promise for treatment of high-risk category medulloblastoma in different subgroups.

Interestingly, *PIK3CA* expression is highest in the SHH subgroup (see Fig. 2A). Also, expression of the HH effector *GLI1* correlates with *PIK3CA* expression and is associated with enrichment of the PI3K-AKT pathway (see Fig. 2B,C). GLI transcription factors are the main downstream effectors of the canonical and the non-canonical HH cascade and are involved in the stimulation of multiple oncogenic signalling pathways^41^. We found nuclear GLI1 protein amounts were reduced in DAOY cells after combined PI3Kα and mTOR inhibition, indicating a role for PI3K/mTOR signalling in the regulation of GLI1 in the SHH subgroup. This observation prompted us to further investigate the mechanistic role of PI3Kα and mTOR in additional HH-driven paediatric cancers.

Survival and tumorigenesis of Ewing sarcoma is dependent on EWS-FLI1, a constitutively active chimeric transcription factor^50^. The HH effector GLI1 is a direct transcriptional target of EWS-FLI1 and exerts key roles in Ewing sarcoma tumorigenesis^40^. We chose Ewing sarcoma because this tumour type was shown to directly dependent on GLI1 and knockdown or inhibition of GLI1 exerts antineoplastic effects in this cancer^40,51,52^. We found that combination of alpelisib and OSI-027 resulted in substantial reduction of GLI1 nuclear protein. This suggests that continuous activity of the PI3K/mTOR axis is required to maintain high levels of GLI1 in the nucleus and inhibition of this pathway results in a reduction of nuclear GLI1 protein amounts. This is in agreement with the finding that the mTOR downstream effector S6K1 directly phosphorylates GLI1, resulting in nuclear translocation and activation of GLI1 independent of SMO^15^. Significantly, Ewing sarcoma cells were highly sensitive to PI3Kα and mTOR inhibition with IC_50_ values in the nanomolar range for the combination of both inhibitors. These findings suggest that PI3K/mTOR signalling contributes to tumorigenesis in HH-driven paediatric cancers through non-canonical activation of HH downstream effectors independently of canonical HH signalling through SMO. They further raise the possibility of targeting the PI3K/mTOR axis as a promising therapeutic approach in HH driven cancers that are resistant to SMO inhibitors. This notion is supported by the finding of the TC71 Ewing Sarcoma cell line showing resistance to the SMO inhibitor cyclopamine^40^, while being highly susceptible to combined PI3Kα and mTOR inhibition in the nanomolar range. This is of particular interest because medulloblastomas resistant to SMO inhibitors have been shown to activate the PI3K/AKT/mTOR pathway^19,20^. Thus it is possible that increased PI3K and mTOR activities contribute to SMO inhibitor resistance by sustaining high nuclear GLI1 levels. In line with this, PI3K/mTOR inhibitors interfere with the development of resistance to SMO inhibitors in medulloblastoma^19^. Hence, PI3Kα/mTOR targeting might be a promising strategy for HH-driven cancers that are resistant to SMO inhibitors.

Here, we demonstrate potent anti-tumor effects after dual PI3K/mTOR inhibition that may be mediated, at least in part, by disrupting nuclear GLI1. Still, given the complexity of the crosstalk between the HH and PI3K/mTOR pathways, it is possible that inhibitory effects on additional pathway components contribute to these antineoplastic effects. Further mechanistic studies will be required to elucidate to which extent the disruption of stem cell frequencies, the inhibition of mTOR mediated mRNA translation and the inhibitory effects on nuclear GLI1 protein contribute to this phenotype. Nevertheless, our findings suggest that HH-driven paediatric cancers can be targeted by inhibition of the PI3K/mTOR pathway and raise the interesting possibility that this strategy might be exceptionally efficient in cancers that are highly dependent on non-canonical HH signalling and that are resistant to SMO inhibitors.

## Materials and Methods

### Cell culture and reagents

DAOY and D556 medulloblastoma cells were grown in DMEM supplemented with 10% FBS and gentamycin (0.1 mg/ml). TC71 and RDES Ewing sarcoma cells were grown in RPMI-1640 supplemented with 10% FBS and penicillin (100 U/ml)/streptomycin (100 ng/ml). 3-D stem-like cancer cell cultures were described in detail previously^26,53,54^. The identity of established cell lines was authenticated by short-tandem repeat (STR) analysis (Genetica DNA Laboratories) in December 2017 (medulloblastoma lines) and August 2016 (Ewing sarcoma lines). All cells were grown at 37 °C in 5% CO_2_. Aleplisib (BYL-719) and OSI-027 were purchased from ChemieTek and dissolved in DMSO as vehicle for *in vitro* studies.

### Immunoblotting and antibodies

Cells were lysed in phosphorylation lysis buffer (pH 7.9) containing 50 mM HEPES, 150 mM NaCl, 1 mM MgCl_2_, 0.5% sodium deoxycholate and supplemented with phosphatase and protease inhibitors (Roche). For cell fractionation, the NE-PER Nuclear and Cytoplasmic Extraction Kit (Thermo Fisher) was used according the manufacturer’s instructions. Protein concentration was quantified by Bradford assay (Bio-Rad) with the Synergy HT plate reader and Gen5 software (Biotek). Equal amounts of protein lysate were subjected to SDS–PAGE (Bio-Rad) followed by transfer to Low Fluorescent PVDF membranes (Bio-Rad). Membranes were blocked with 5% BSA or 5% milk and cut horizontally to allow for incubation with different primary antibodies overnight at 4 °C. Membranes were then incubated in secondary horseradish peroxidase (HRP)-conjugated antibody for 1 hour, and analysed using the WesternBright ECL substrate (GE Healthcare) and either autoradiography film (Denville Scientific) or the ChemiDoc MP Imaging System and Image Lab 5.0 software (Bio-Rad) as described^26^. Antibodies were removed, where indicated, using Restore PLUS Western Blot Stripping Buffer (Thermo Fisher) to allow for incubation with additional antibodies. A comprehensive list of antibodies used in this study can be found in Supplemental Table 1. Uncropped blots are presented in the supplement.

### Cell viability and apoptosis assays

Cell viability was assessed using the Cell Proliferation Reagent WST-1 (Roche) as described previously^26^. Briefly, cells were seeded into 96-well plates at a density of 1,500 cells per well (for medulloblastoma cell lines) or 5,000 cells per well (Ewing sarcoma cell lines) with the indicated drugs. After incubation at 37 °C in 5% CO_2_ for 5 days, the WST-1 reagent (10% v/v) was added and cell viability was assessed using the Synergy HT plate reader and Gen5 software (BioTek) as described^55^. Apoptosis assay was done as described before^56^.

### Soft agar assays

For investigation of anchorage-independent cell growth, soft-agar assays were performed using the CytoSelect 96-Well Cell Transformation Assay Kit (Cell Biolabs, Inc.) according to the manufacturer’s instructions. In brief, medulloblastoma and Ewing sarcoma cells were seeded in soft-agar in a 96-well plate and incubated at 37 °C in 5% CO_2_ with the indicated inhibitors for 7 days (medulloblastoma cell lines) or 10 days (Ewing sarcoma cell lines) before agar was solubilized and cells were lysed according to the manufacturer’s instructions. Colony formation was assessed as previously described^55^.

### Bioinformatics and statistical analysis

For gene expression analysis, the GlioVis portal^57^ was employed to obtain data of the Northcott_2012 dataset^39^. These data were subjected to analysis in GraphPad Prism 7.0 as described before^26^. Also, statistical analyses were performed using Prism Graphpad 7.0, including calculation of IC_50_ values. For comparison of more than two groups one-way analysis of variance (ANOVA) was used followed by Tukey’s test. For comparison of more than two groups and multiple time points, two-way ANOVA was used followed by Tukey’s test. Comparison of stem cell frequencies of different groups for ELDA was done by Chi square (χ^2^) test. CI values were calculated for the IC_50_ values using Compusyn as described before^58^.

### siRNA-mediated knockdown of gene expression

*PIK3CA* and control siRNA were from Dharmacon (GE Healthcare) and used with the Lipofectamine RNAiMAX Reagent and the Opti-MEM medium (Thermo Fisher) as described previously^26^.

### Neurosphere assay and extreme limiting dilution analysis (ELDA)

DAOY and D556 cells were seeded at indicated cell concentrations in round-bottom 96-well plates (Costar) containing serum free media and the indicated drugs and incubated for one week at 37 C in 5% CO_2_. After 1 week, spheres were stained with acridine orange as previously described^54^. Spheres for neurosphere assay were imaged as described in^26^ and spheres for ELDA were imaged as described previously^54^. Extreme limiting dilution analysis (ELDA) was carried out as previously described^26^ using the ELDA online software (http://bioinf.wehi.edu.au/software/elda/).

### DAOY flank tumour xenograft study

Mouse studies were carried out in accordance with approved protocols by Institutional Animal Care and Use Committee (IACUC) at Northwestern University. Five to 6-week-old athymic nude female mice (NU/NU strain 088; Crl:NU-*Fox1*^*nu*^) were purchased from Charles River and housed under aseptic conditions. Flank tumours were established by subcutaneous injection of 5 × 10^6^ cells in Matrigel (Corning). Mice were monitored and tumours were measured by calliper as described before^26^. For *in vivo* studies, alpelisib was dissolved in Ora-Plus and OSI-027 was dissolved in 20% Trappsol in water and both drugs were administered by oral gavage. Mice were treated with (i) vehicle control (VC); (ii) alpelisib (50 mg/kg); (iii) OSI-027 (25 mg/kg); or (iv) the combination of alpelisib (50 mg/kg), and OSI-027 (25 mg/kg).

## Supplementary information

Supplemental Information

## Data Availability

Datasets and analysis tools used in this study can be accessed from the GlioVis portal (http://gliovis.bioinfo.cnio.es/).
