# Supplementary material for: Pharmacological mTOR targeting enhances the antineoplastic effects of selective PI3Kα inhibition in medulloblastoma
Source: Sci Rep. 2019 Sep 6;9:12822. doi: 10.1038/s41598-019-49299-3 (PMC6731286; doi:10.1038/s41598-019-49299-3)

Pharmacological mTOR targeting enhances the antineoplastic effects of selective PI3Kα inhibition in medulloblastoma

Frank Eckerdt[1,2,§,*], Jessica Clymer[1,3,§,¶], Jonathan B. Bell[1], Elspeth M. Beauchamp[1,4,5], Gavin T. Blyth[1], Stewart Goldman[1,3], and Leonidas C. Platanias[1,4,5]

[1]Robert H. Lurie Comprehensive Cancer Center of Northwestern University, Chicago, IL, USA

[2]Department of Neurological Surgery, Feinberg School of Medicine, Northwestern University, Chicago, IL, USA

[3]Division of Hematology/Oncology/Neuro Oncology/Stem Cell Transplantation, Department of Pediatrics, Ann & Robert H. Lurie Children's Hospital of Chicago, Chicago, IL, USA

[4]Division of Hematology/Oncology, Department of Medicine, Feinberg School of Medicine, Northwestern University, Chicago, IL, USA

[5]Medicine Service, Jesse Brown VA Medical Center, Chicago, IL, USA

[§]Both authors contributed equally

[¶]Current address: Dana-Farber/Boston Children's Cancer and Blood Disorders Center, Boston, MA, USA

[*]Correspondence to: Frank Eckerdt, Robert H. Lurie Comprehensive Cancer Center,
303 East Superior Street, Lurie 3-220, Chicago, IL 60611, Tel. 312-503-0292;
Email: frank.eckerdt@northwestern.edu

Supplemental Table 1

ANTIBODY	SOURCE	IDENTIFIER
p70S6K (49D7)	Cell Signaling Technology	Cat#:2708 RRID:AB_390722
p-p70S6K T389	Cell Signaling Technology	Cat#:9205 RRID:AB_330944
Lamin A/C	Cell Signaling Technology	Cat#:2032 RRID:AB_2136278
Beta-tubulin (D3U1W)	Cell Signaling Technology	Cat#:86298 RRID:AB_2715541
Alpha-tubulin (B-7)	Santa Cruz Biotechnology	Cat#:sc-5286 RRID:AB_628411
HSP-90 (H-114)	Santa Cruz Biotechnology	Cat#:sc-7947 RRID:AB_2121235
PI3 Kinase p110alpha (c73F8)	Cell Signaling Technology	Cat# 4249 RRID: AB_2165248
GAPDH	Millipore	Cat# MAB374, RRID:AB_2107445
Pan AKT (40D4)	Cell Signaling Technology	Cat# 2920 RRID:AB_1147620
p-AKT S473 (D9E)	Cell Signaling Technology	Cat# 4060 RRID:AB_2315049
4EBP1	Cell Signaling Technology	Cat# 9452 RRID:AB_331692
p-4EBP1 T37/46	Cell Signaling Technology	Cat# 9459 RRID: AB_330985
GLI1	Cell Signaling Technology	Cat# 3538 RRID:AB_1903989
HDAC1 (10E2)	Cell Signaling Technology	Cat# 5356 RRID:AB_10612242
Rabbit IgG HRP Linked Whole Ab	GE Healthcare	NA934-1ML RRID:AB_772206
Goat Anti-Mouse IgG (H + L)-HRP Conjugate	Biorad	Cat#:1706516 RRID:AB_11125547

Supp Table 1: List of antibodies used in this study, their manufacturers, catalog numbers and Research Resource Identifier (RRID) numbers.

Figure 1A and B full blots (films)

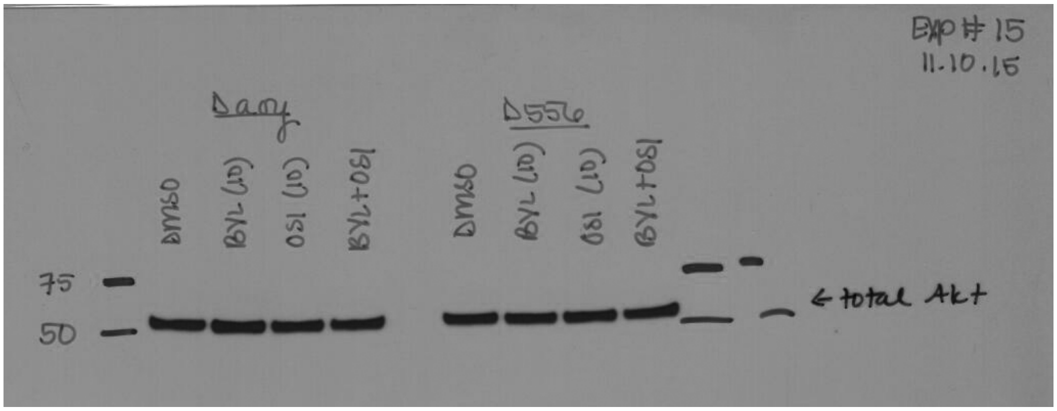

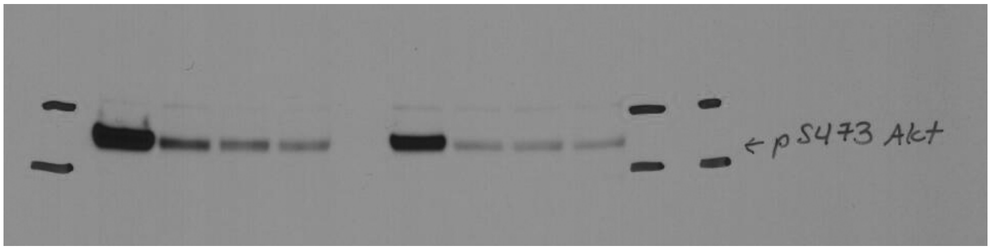

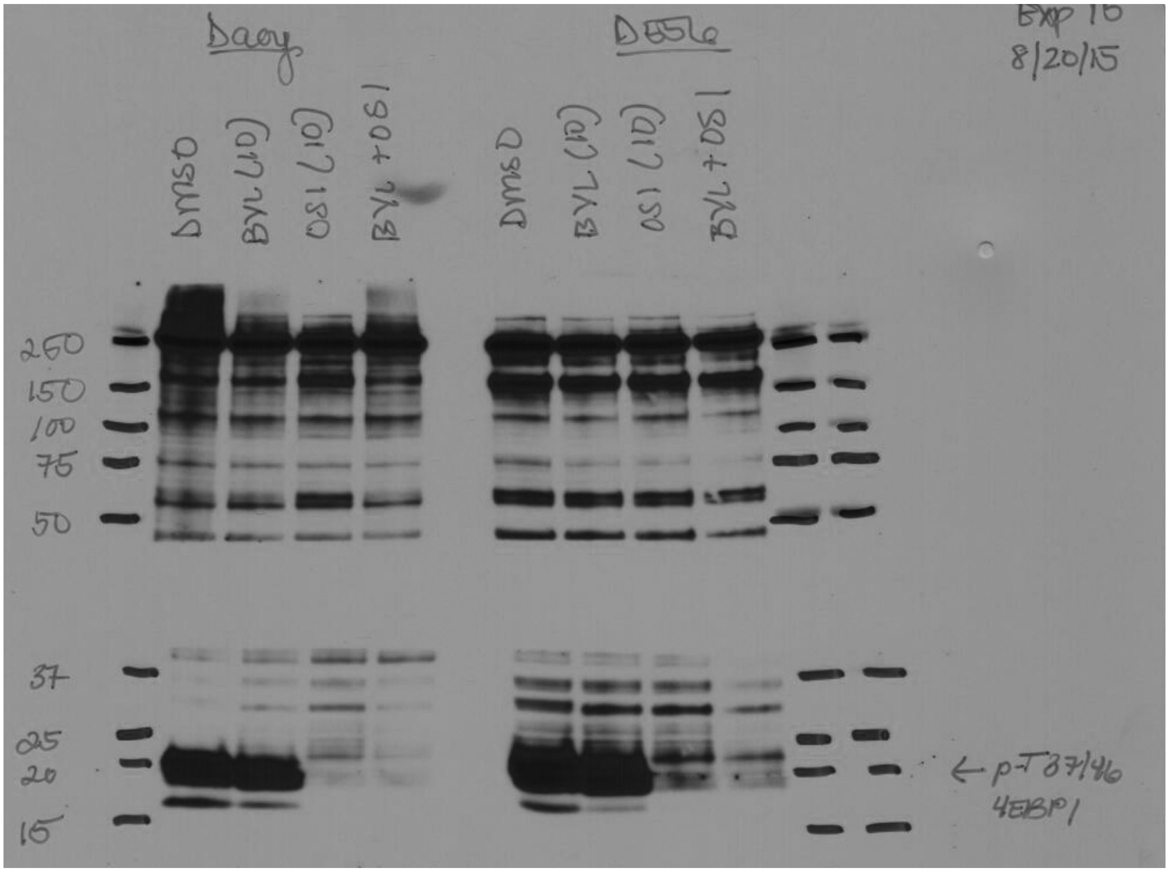

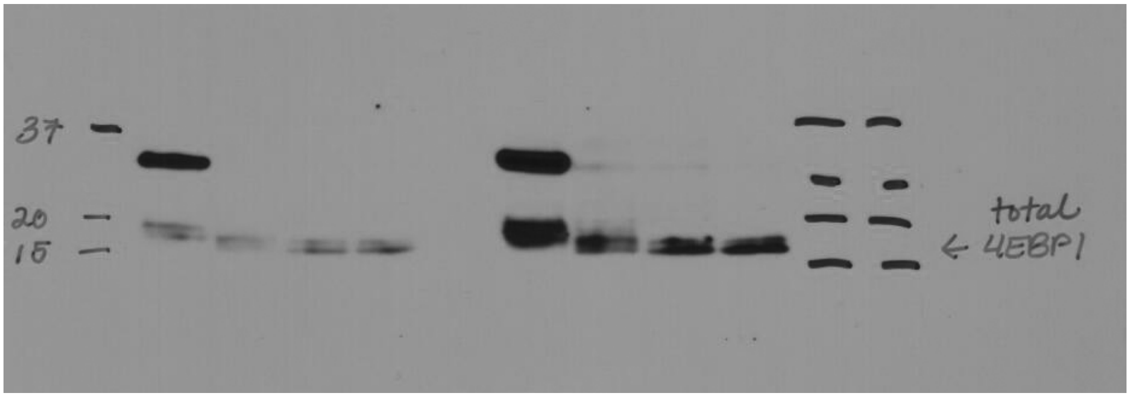

Figure 1A and B full blots (films)

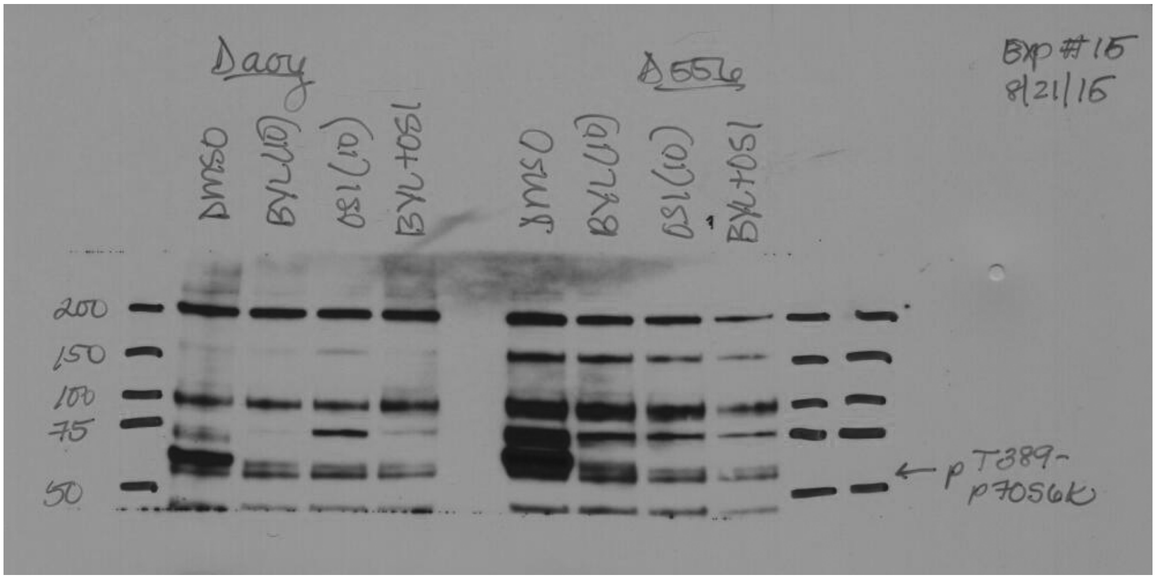

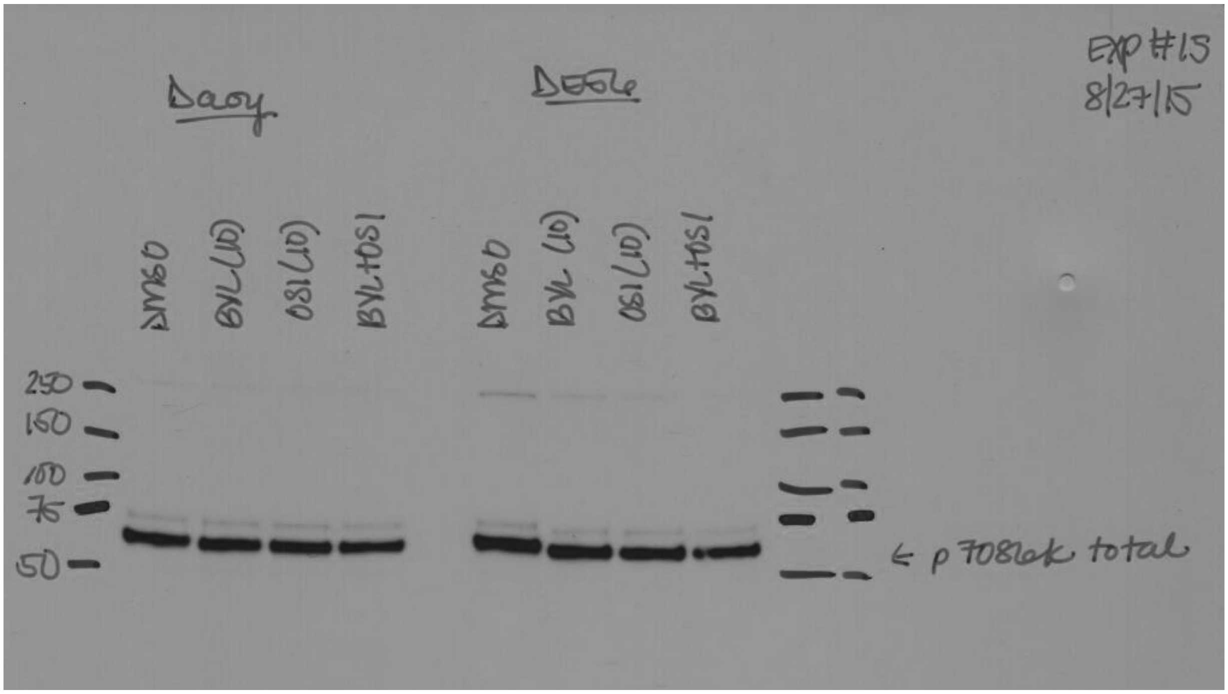

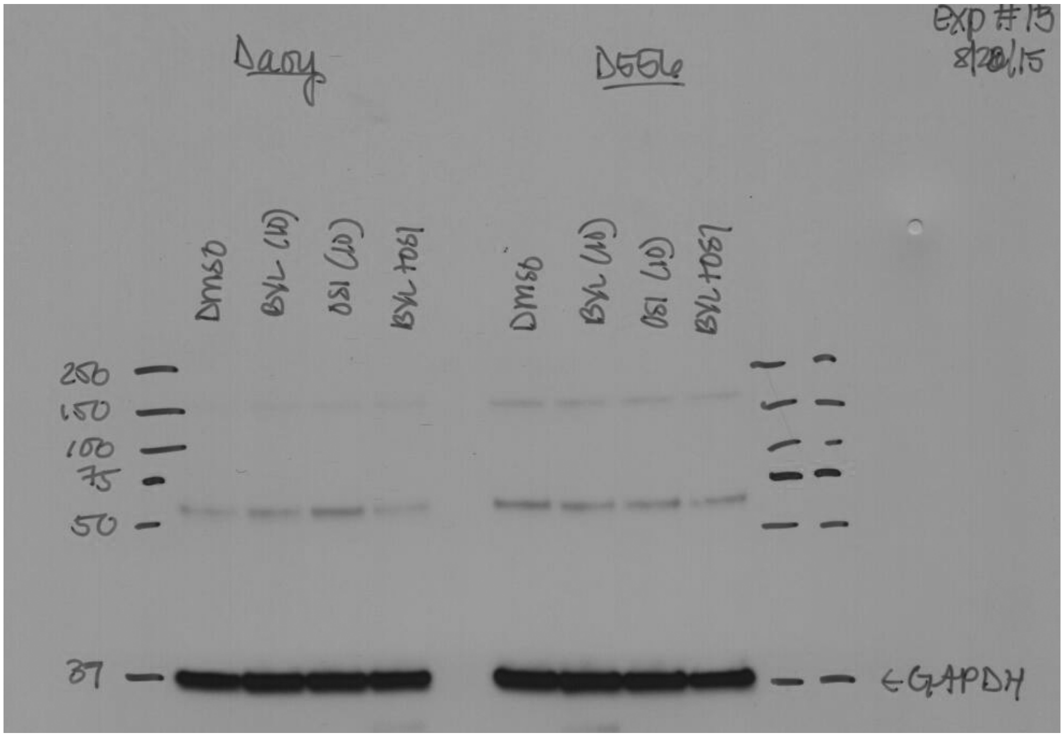

Figure 3A full blots (Biorad Chemidoc)

11.3 sec exposure

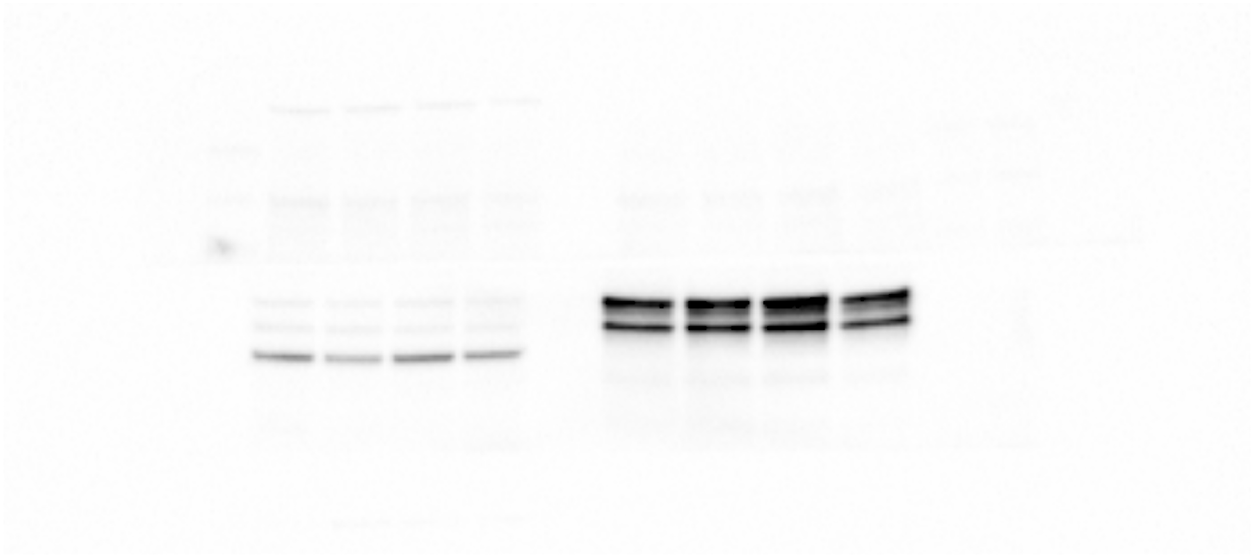

GLI1

LaminA/C
and tubulin

289.7 sec exposure

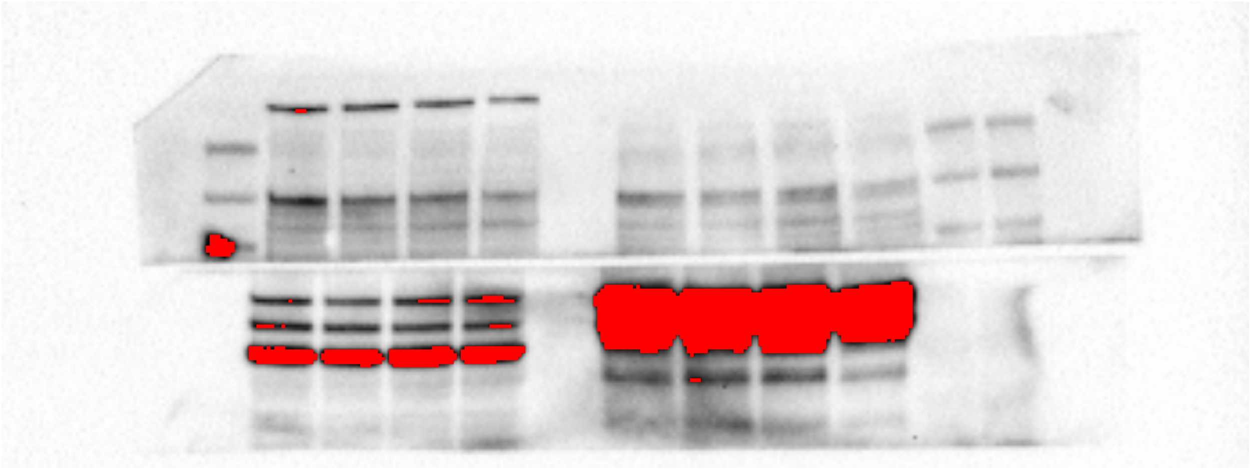

GLI1

LaminA/C
and tubulin

Figure 3B full blots (Biorad Chemidoc)

5.1 sec exposure

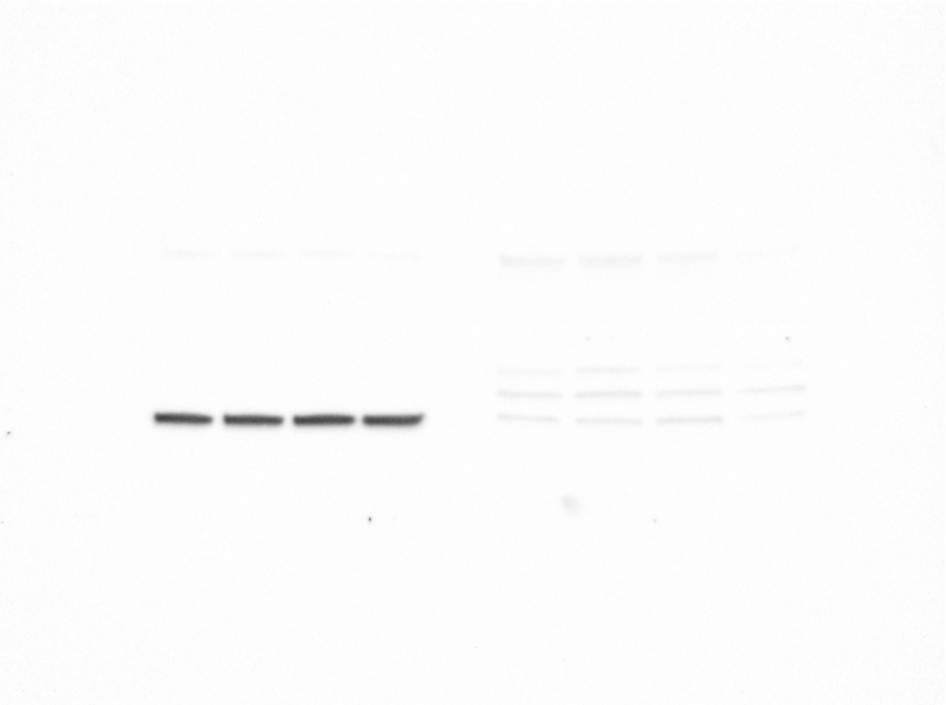

120.0 sec exposure

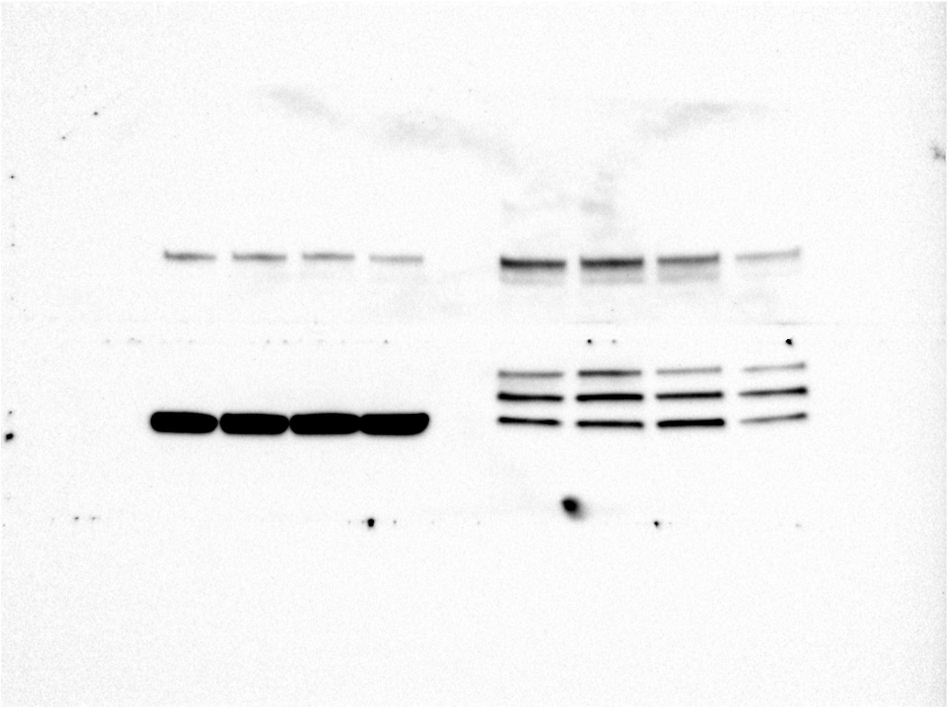

Figure 3C full blots (Biorad Chemidoc)

120.0 sec exposure

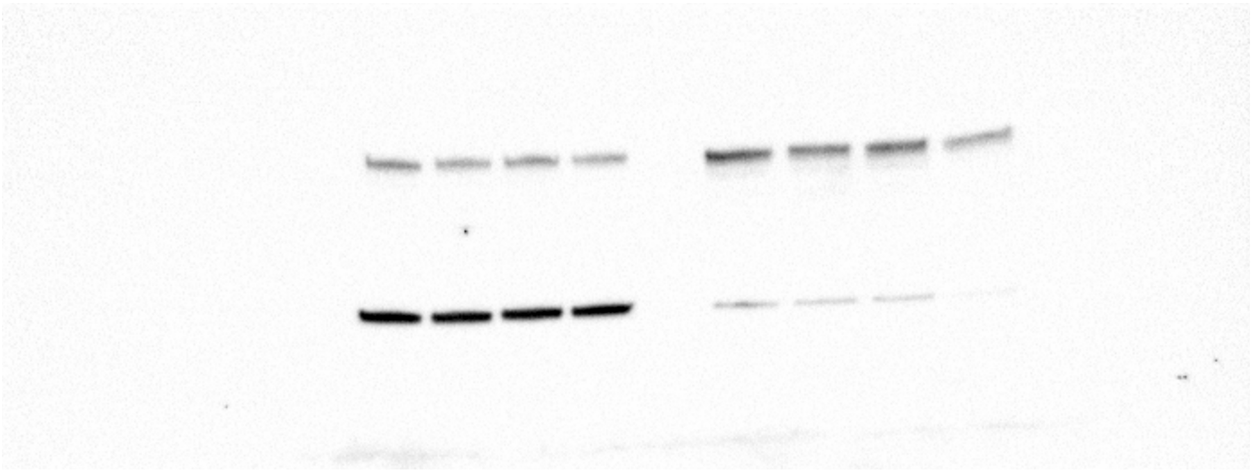

GLI1

tubulin

120.0 sec exposure

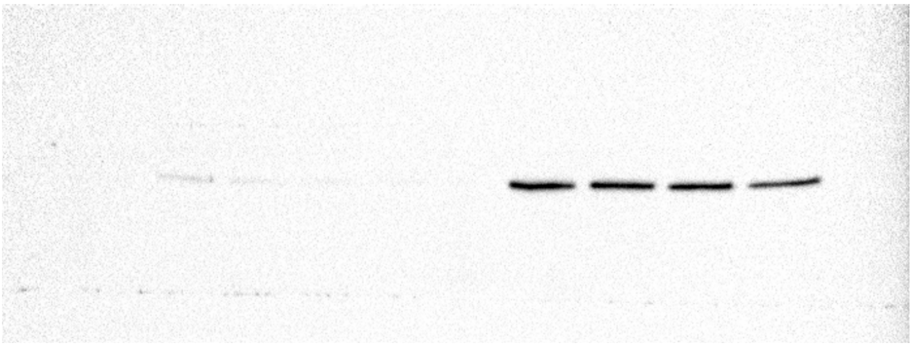

HDAC1

Figure 4G and H full blots (films).
Other p110 isoform siRNAs were cropped out because a similar experiment has been published in our previous study (ref. 26).

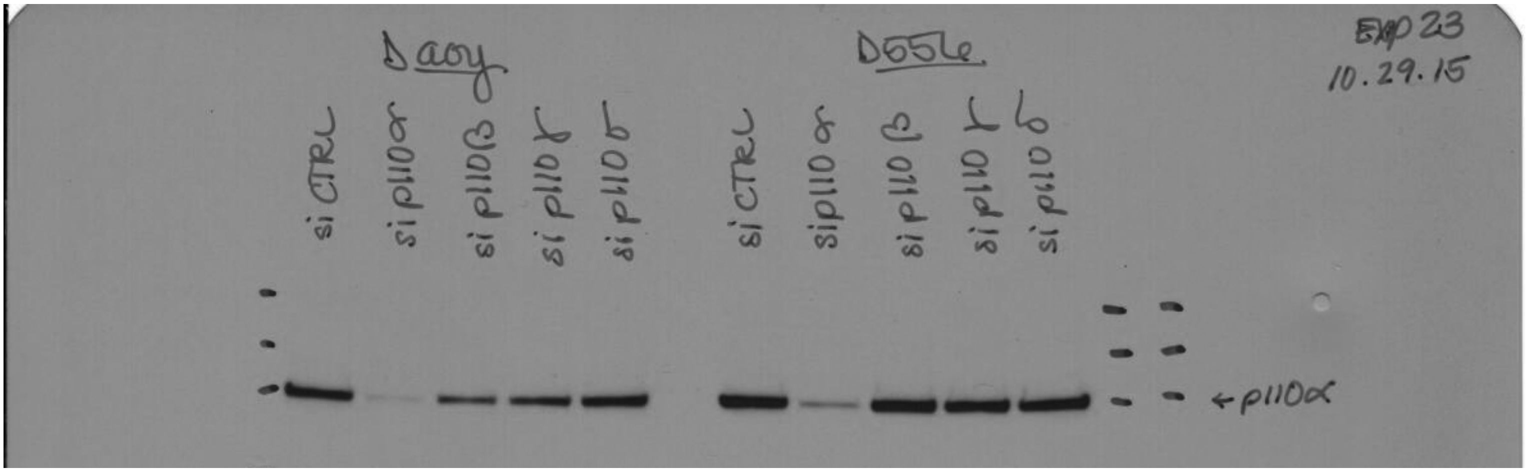

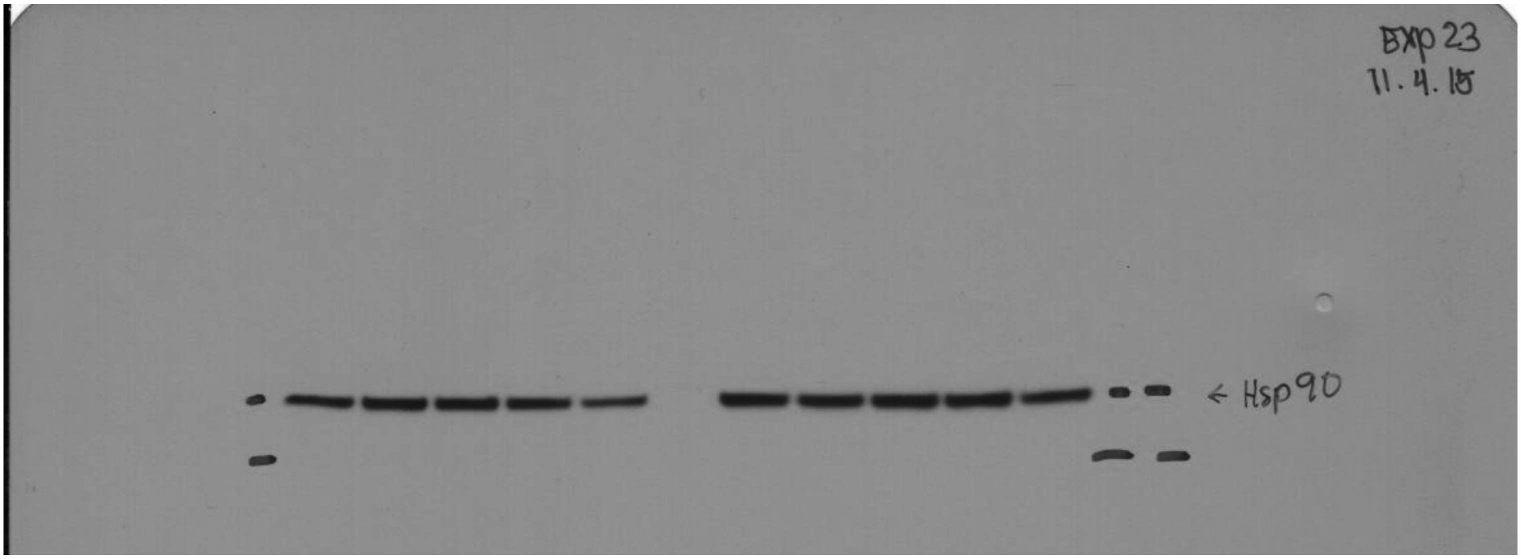

Supplement: Supplementary file 1 — Supplemental Information [file 41598_2019_49299_MOESM1_ESM.pdf]
